# PANCREATODUODENECTOMY DUE TO LIPOMATOUS PSEUDOHYPERTROPHY OF THE PANCREAS

**DOI:** 10.1590/0102-672020230036e1754

**Published:** 2023-09-15

**Authors:** Orlando Jorge Martins Torres, Rodrigo Rodrigues Vasques, Cláudio Matias Barros, Galvani Ascar Sauaia, Benedito Dario Murad Mouchrek, Marcelo Lima Rocha, Rennan Abud Pinheiro Santos, Milena Vasconcelos Falcão, José Maria Assunção Moraes

**Affiliations:** 1 Universidade Federal do Maranhão, Department of Gastrointestinal Surgery, Hepatopancreatobiliary Unit – São Luís (MA), Brazil; 2 Hospital São Domingos, Department of Radiology – São Luís (MA), Brazil.

**Keywords:** Pancreatic Diseases, Lipomatosis, Pancreatoduodenectomy, Pancreatopatias, Lipomatose, Duodenopancreatectomia

## Abstract

**BACKGROUND:**

Lipomatous pseudohypertrophy of the pancreas, pancreatic lipomatosis, pancreatic steatosis, non-alcoholic fatty pancreatic disease, or fatty pancreas is an extremely rare disease, characterized by the organ enlargement and a localized or diffuse replacement of pancreatic acinar cells by mature adipose tissue, preserving the pancreatic ductal system and islets of Langerhans.

**AIMS:**

To report a rare case of lipomatous pseudohypertrophy of the pancreas in a symptomatic patient and the surgical treatment employed.

**METHODS:**

A 24-year-old male patient with weight loss (10 kilograms in 8 months), hyperglycemia, severe and recurrent acute abdominal pain, epigastric discomfort associated with nausea, vomiting, and jaundice for 40 days. Magnetic resonance imaging was performed, revealing an irregular lipomatous pseudohypertrophy of the pancreas, measuring 6.0 × 5.6 cm in the head, uncinate process, and part of the body of the pancreas. The pancreatic duct dilation was diffuse and irregular, associated with atrophy of the remnant parenchyma, particularly in the tail of the pancreas. The patient underwent pancreatoduodenectomy without total mesopancreas excision followed by pancreatojejunostomy.

**RESULTS:**

The postoperative course was uneventful, the length of stay in the ICU was two days, and the patient was discharged on the seventh postoperative day.

**CONCLUSIONS:**

The disease treatment depends on the signs and symptoms at presentation and a pancreatoduodenectomy is indicated in patients with severe and recurrent abdominal pain.

## RESUMO

**RACIONAL:**

Pseudo-hipertrofia lipomatosa do pâncreas, lipomatose pancreática, esteatose pancreática, doença pancreática gordurosa não alcoólica ou pâncreas gorduroso é uma doença extremamente rara, caracterizada por aumento do órgão e substituição localizada ou difusa de células acinares pancreáticas por tecido adiposo, preservando o sistema ductal pancreático e as ilhotas de Langerhans.

**OBJETIVOS:**

Relatar um caso raro de pseudo-hipertrofia lipomatosa do pâncreas, em paciente sintomático e o tratamento cirúrgico empregado.

**MÉTODOS:**

Paciente do sexo masculino, 24 anos, com queixas de emagrecimento (10 quilos nos últimos 8 meses), hiperglicemia, dor abdominal aguda intensa e recorrente, desconforto epigástrico associado a náuseas, vômitos e icterícia há 40 dias. A ressonância magnética (RM) revelou pseudo-hipertrofia lipomatosa irregular do pâncreas, medindo 6,0 × 5,6 cm na cabeça, processo uncinado e parte do corpo do pâncreas. A dilatação do ducto pancreático era difusa e irregular, associada à atrofia do parênquima remanescente, principalmente na cauda do pâncreas. O paciente foi submetido à duodenopancreatectomia sem excisão total do mesopâncreas seguida de pancreatojejunostomia.

**RESULTADOS:**

O pós-operatório transcorreu sem intercorrências, o tempo de internação na UTI foi de 2 dias, com alta hospitalar no sétimo dia.

**CONCLUSÕES:**

O tratamento da doença depende dos sinais e sintomas na apresentação e a duodenopancreatectomia é indicada em pacientes com dor abdominal intensa e recorrente.

## INTRODUCTION

Lipomatous pseudohypertrophy (LPH) of the pancreas, also known as pancreatic lipomatosis, pancreatic steatosis, non-alcoholic fatty pancreatic disease, or fatty pancreas is an extremely rare disease, characterized by the organ enlargement and the localized or diffuse replacement of pancreatic acinar cells by mature adipose tissue, preserving the pancreatic ductal system and islets of Langerhans^1,3^. Pancreatic lipomatosis has no single etiology, and it seems to be related to viral infection, ductal obstruction, toxin exposure, or advanced cystic fibrosis^1,3^. An association with some rare childhood syndromes as cystic fibrosis, Bannayan syndrome, Shwachman-Diamond syndrome, or Johanson-Blizzard syndrome has been reported^1,6,8,12^. Major pancreatic LPH is limited and have no clinical significance. However, in patients with severe fatty replacement, a significant depression of the pancreatic function has been observed, which may cause exocrine pancreatic insufficiency^3,8^. The most common clinical symptoms associated with this condition are chronic diarrhea, weight loss, steatorrhea, diabetes and maldigestion of nutrients^3,11^. Abdominal pain is not common in these patients.

This study aimed at reporting a rare case of LPH of the pancreas in a symptomatic patient and the surgical technique employed.

## CASE REPORT AND SURGICAL TECHNIQUE

A 24-year-old male patient presented to the hospital complaining of weight loss (10 kilograms in the last 8 months), hyperglycemia, severe and recurrent acute abdominal pain, epigastric discomfort associated with nausea, vomiting, and jaundice for 40 days. The patient was admitted to the emergency unit for hyperglycemia control, and diabetes mellitus was confirmed. Malabsorption syndrome associated with weight loss and chronic diarrhea (six or more greasy stools/day) was not related in the present study. Moreover, no episodes of fever, and no history of alcoholism were reported. Respiratory and cardiovascular system examination revealed no abnormalities, and his familial history was normal. Abnormal laboratory test results were gamma-glutamyl transpeptidase (197 U/L) and glycated hemoglobin (9,7%). On admission, serum pancreatic amylase and lipase levels, serum total cholesterol, low-density lipoprotein cholesterol, high-density lipoprotein cholesterol, and triglyceride levels were normal.

Physical examination revealed a palpable abdominal mass in the topography of the upper right abdomen and mild tenderness on deep palpation. The patient underwent an abdominal ultrasound that revealed an increase in the volume of the pancreatic head, compressing the duodenum, a dilated pancreatic duct, and biliary sludge inside the gallbladder.

**Figure 1:**
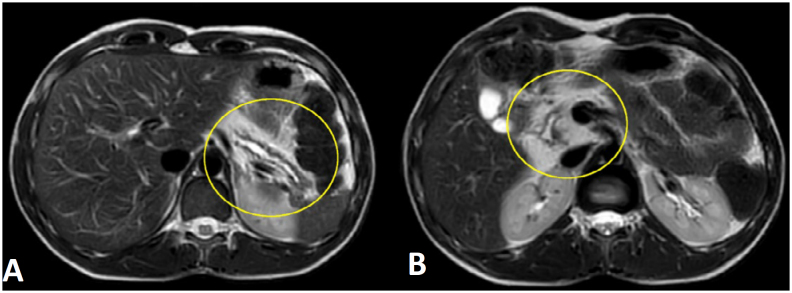
(**A**) T2-weighted axial image without fat signal saturation. Identification of atrophy of the pancreatic tail, ductal dilation, and homogeneous peripancreatic fat. (**B**) Enlargement of the head and part of the body of the pancreas by fat tissue (hyperintense).

**Figure 2:**
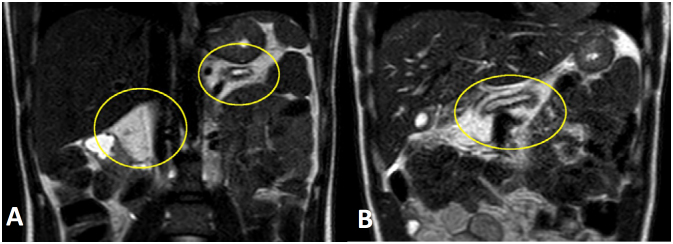
(**A** and **B**) T2-weighted axial images without fat signal saturation. The pancreatic parenchyma is replaced by adipose tissue.

Magnetic resonance imaging was also performed, revealing an irregular LPH of the pancreas, measuring 6.0 × 5.6 cm in the head, uncinate process, and part of the body of the pancreas. The pancreatic duct dilation was diffuse and irregular, associated with atrophy of the remnant parenchyma, particularly in the tail of the pancreas (Figures 1 and 2).

Based on these clinical findings, and imaging features, the final diagnosis for this patient was LPH of the pancreas. The case was discussed in a multidisciplinary meeting and pancreatoduodenectomy was indicated.

**Figure 3:**
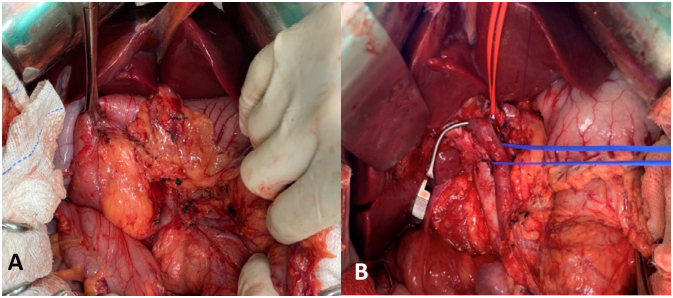
(**A**) Pancreatic head replaced by adipose tissue. (**B**) Mobilization of the pancreatic head, and isolated portal vein.

**Figure 4:**
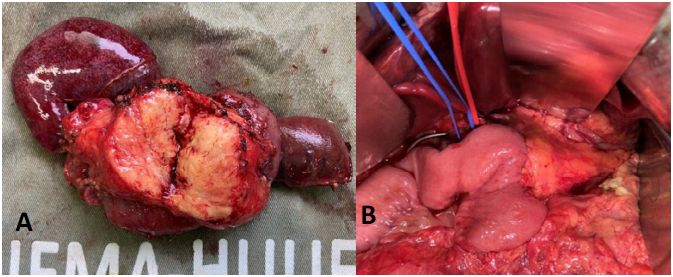
(**A**) Pancreatic head replaced by adipose tissue. (**B**) Pancreatic anastomosis.

After a complete preoperative evaluation, the patient underwent pancreatoduodenectomy without total mesopancreas excision. Pancreatojejunostomy was performed according to our classic technique^4,13^ (Figures 3 and 4). The postoperative course was uneventful, the length of stay in the ICU was two days, and the patient was discharged on the seventh postoperative day. The pathology of the specimen confirmed the replacement of pancreatic parenchyma by adipose tissue. After six months of follow-up the patient was asymptomatic and receiving pancreatic enzymes replacement therapy regularly. The patient agreed and signed the informed consent for this report.

## DISCUSSION

LPH of the pancreas is a rare entity characterized by the near-complete absence of pancreatic exocrine tissue, replacement of the parenchyma by adipose tissue, and an increase in pancreatic size and weight with pseudotumor formation. With the expansion in volume of the pancreas, the pancreatic exocrine tissue is almost completely absent and replaced by fat tissue, preserving the system of ducts and islets of Langerhans^1,3,8^. The involvement of the pancreas can be diffuse or located only on the head, body, or tail. Because of the enlarged pancreas, especially the head, the duodenum can be compressed, leading to abdominal discomfort at presentation^3,7,8^. LPH has no effect on the endocrine function of the pancreas; however, when it is massive, it can be associated with exocrine pancreatic insufficiency, with steatorrhea, and malabsorption of fat-soluble vitamins^3,6,8,10,11^. In the present study, the patient was admitted for hyperglycemia control. Diabetes mellitus was confirmed as well as a possible association with the disease.

LHP was first reported by Hantelmann in 1931^5^ and its etiology remains unknown. However, various hypotheses have been suggested. It seems to be associated with viral infection, ductal obstruction, toxin exposure, advanced cystic fibrosis, some specific syndromes, or may be caused by abnormal metabolism. Because it forms a mass, it is frequently mistaken for a malignant disease.

Ultrasonography is very limited for the diagnosis of LPH. Endoscopic ultrasound–guided and fine-needle aspiration biopsy might be helpful in some cases. On computed tomography image, the pancreas appears increased in size and the parenchyma appears infiltrated or replaced by a tissue with fat-density component, which is similar to retroperitoneal fat^1–3,14^.

In the present case, the magnetic resonance imaging scan showed pancreatic enlargement, fat infiltration, and atrophy of the parenchyma, particularly T1-weighted and T2-weighted imaging with diffuse and homogeneous T1 and T2 hypersignals replacing the pancreatic parenchyma with signal suppression on fat saturation sequences. The pancreatic parenchyma was preserved and the apparent diffusion coefficient (ADC) value was normal. On the magnetic resonance cholangiopancreatography (MRCP) images, the pancreas ductal system was normal.

Macroscopically, the appearance and consistency of the pancreas are those of mature adipose tissue. Microscopically, it has well-defined borders without a well-formed capsule and a massive replacement of the parenchyma by adipose tissue^3,9,10^. The islets of Langerhans are relatively preserved, there is admixture of normal pancreatic parenchyma within the lesion and no significant inflammation, fibrosis or necrosis^1,3^. These pancreatic elements do not show any signs of either acute or chronic pancreatitis. To obtain a definitive diagnosis, LPH must be distinguished from pancreatic carcinoma and liposarcoma^1,3,8,9^.

There is not an established disease-specific treatment for LPH; however, for patients with exocrine function insufficiency, pancreatic enzymes are administered, combined with a balanced diet, avoiding fatty foods. Some patients have undergone surgical resection due to mass effect and abdominal pain or suspicion of malignant disease^4,10^. In the present study, acute abdominal pain was the most relevant symptom and the decision for pancreatoduodenectomy was taken after other treatments failed.

## CONCLUSIONS

LPH of the pancreas is a very rare disease, with a variety of possible etiologies. The clinical presentation is related to the mass effect and exocrine insufficiency, and in some cases the mass may suggest carcinoma. Computed tomography and magnetic resonance imaging are reliable and effective methods for diagnosis. The treatment depends on the signs and symptoms at presentation and a pancreatoduodenectomy is indicated in patients with severe and recurrent abdominal pain.
